# Working Mechanisms of Exposure and Response Prevention in the Treatment of Tourette Syndrome and Tic Disorders Revisited: No Evidence for within-Session Habituation to Premonitory Urges

**DOI:** 10.3390/jcm12227087

**Published:** 2023-11-14

**Authors:** Jolande M. T. M. van de Griendt, Nelleke M. E. van den Berg, Cara W. J. Verdellen, Daniëlle C. Cath, Marc J. P. M. Verbraak

**Affiliations:** 1 Behavioural Science Institute, Radboud University Nijmegen, Thomas van Aquinostraat 4, 6525 GD Nijmegen, The Netherlands; m.verbraak@propersona.nl; 2 Department of Psychology, Erasmus University Rotterdam, ‘s Gravendijkwal 230, 3015 CE Rotterdam, The Netherlands; nellekeberg@solcon.nl; 3 PsyQ Nijmegen, Parnassia Group, Sint Annastraat 263, 6525 GR Nijmegen, The Netherlands; c.verdellen@psyq.nl; 4 Department of Psychiatry, University Medical Center Groningen, Rijksuniversiteit Groningen, 9700 AD Groningen, The Netherlands; d.c.cath@umcg.nl; 5 GGZ Drenthe, Dennenweg 9, 9404 LA Assen, The Netherlands; 6 Pro Persona, Wolfheze 2, 6874 BE Wolfheze, The Netherlands

**Keywords:** behavior therapy, exposure and response prevention (ERP), premonitory urge, habituation, Tourette syndrome

## Abstract

Background: Exposure and response prevention (ERP) has been shown to be an effective treatment for Tourette syndrome (TS) and chronic tic disorders (CTD). ERP is based on voluntary tic suppression in combination with prolonged exposure to premonitory urges preceding tics. A prevailing hypothesis of the working mechanism underlying ERP in tics is habituation to the premonitory urges as a result of prolonged exposure. However, results so far are equivocal. This study aims to further explore the relation between urges and ERP in tics, by investigating the course of premonitory urges during ERP sessions. Methods: Using a data-driven approach, within-session habituation to premonitory urge intensity was investigated. In total, 29 TS patients rated urge intensity at seven timepoints during ten 1 h ERP sessions. Results/Conclusions: Latent growth modeling showed an increase in urge intensity during the first 15 min of each session followed by a plateau in the remaining 45 min of the session. This does not support the idea of within-session habituation to premonitory urges as a working mechanism of ERP. Other potential underlying working mechanisms are discussed and should be tested in future research.

## 1. Introduction

Tourette syndrome (TS) is a neuropsychiatric disorder characterized by multiple simple or complex, motor and vocal tics [1]. In most cases, two essential “tic features” can be recognized that are closely associated, i.e., an inner tension that accompanies tic suppression, and the feeling of active involvement in performing a tic, especially in adults [2]. Although patients are unable to continuously and voluntarily suppress their tics, they may experience the tic as a conscious, intentional and self-directed movement executed to relieve a so-called “premonitory urge” or “premonitory sensation” [3,4]. This feeling of intentionality increases with age, and by age 12 the majority of patients recognize a premonitory urge preceding a tic [5,6]. This sensation is somatosensory in nature [6,7,8,9] and can manifest itself for example in the skin, muscles, joints, bones or vocal cords. The premonitory urge can involve the experience of pressure, energy, pain or itch. After the manifestation of the tic, there is temporary relief or a reduction in the sensation [3]. The premonitory urge can be considered the involuntary component of the tic, while the tic—executed in response to the unpleasant premonitory urge—is more intentional in character [3,7,8]. In line with the negative reinforcement theory [10,11,12], it has been hypothesized that as the awareness of these urges increases with age in persons with TS [5], the association between the urge to perform a tic and the tic that relieves the urge itself is expected to be strengthened.

Behavior therapy for tics, especially habit reversal training (HRT) and exposure and response prevention (ERP), specifically aims to interrupt the negative reinforcement cycle via voluntary tic suppression, in combination with exposure to premonitory urges. HRT mainly consists of increasing awareness of a single tic and then practicing competing responses upon the presence of premonitory urges to inhibit the tic. The development of ERP has been inspired by the gold-standard treatment for obsessive compulsive disorder (OCD), where patients suppress their compulsions (e.g., ritualized hand washing), while being exposed to feared stimuli (dirty hands) [13]. Behavior therapy is considered a first-line intervention for tic disorders [14,15,16]. Most evidence is currently available for HRT, which has been investigated in children and adults from 1973 onwards [15,17,18,19,20,21,22]. Although much less investigated, ERP is regarded as an effective treatment for tics in both children and adults as well [22]. Both for ERP as well as for HRT, different treatment modalities have been developed to optimize the treatment. For example, treatment can take place online [23,24,25,26,27,28,29], in groups [21,30,31], for very young children [32], and carried out by different professionals (e.g., nurse practitioners [33] and occupational therapists [34]). Research has been conducted on the duration and spacing of sessions; behavior therapy also works in fewer sessions [35], in intensified programs [36], and in sessions of a shorter duration (1 h ERP instead of 2 h) [37]. The current study is based on the data of patients who underwent 1 h ERP sessions. A significant tic reduction (*p* < 0.001) with an effect size of 0.96 was found; the mean YGTSS total tic scores decreased from 18.8 (SD 7.0) at baseline, to 12.2 (SD 6.8) after treatment with shorter exposure sessions.

Habituation to premonitory urges has been suggested to be a central working mechanism underlying exposure therapy in tics [38]. This model is derived from the original model of anxiety habituation in exposure therapy for anxiety disorders, in which the habituation curve has an inverted U-shape with respect to the course of within-ERP session anxiety severity [39,40]. Initially, anxiety levels increase within a session (also known as “initial fear activation”) [41] followed by a decrease over time (within-session habituation). In tic disorders, according to self-reports and corroborated in experimental settings, sensory sensations and urges are assumed to intensify with tic suppression, until they are relieved via tic performance [11,12,42,43,44]. Hypothetically, after performing the tic, the urge fades out, until it rises again for the next tic, and the cycle repeats itself. Thus, the association between the premonitory urge and the tic is reinforced and becomes stronger. In ERP treatment, the patient learns to prevent tics from occurring while the therapist encourages consciously focusing attention on all sensory aspects of the premonitory urges, while at the same time not giving in to the urge to perform the tic. Theoretically, by carrying this out for a prolonged period of time, the intensity of urges will reach a peak. In the process of habituation, one would expect a decline after this peak, and with training and practice, over time, the amplitude of the habituation–curve is likely to decrease, leading to a decline in urges overall.

Several studies have examined the course of habituation during tic suppression [38,45,46]. Verdellen et al. (2008) [38] tested the habituation hypothesis as a possible underlying mechanism of ERP in 20 TS patients (age 7–55 years) during 10 two-hour sessions. Preceding each session and in 15 min intervals, the urge intensity preceding the four most frequent or interfering tics at that time was rated, as well as the global tension, using the Subjective Unit of Distress Scale ranging from 0 (absent) to 4 (very severe). Although Verdellen et al. (2008) expected an inverted U-shaped habituation curve, they found a linear reduction in urges, both within and between sessions during ERP [38]. In a second study, Specht and colleagues examined, in one 40 min session, the ability of 12 children and adolescents (aged 10–17 years) with TS to suppress tics for prolonged periods [46]. Within the session, tic suppression periods were interspersed with periods in which patients were free to perform tics. Premonitory urge intensity was measured in 10 s intervals. Consistent with Verdellen et al. (2008) [38], there was no significant initial increase in premonitory urge ratings during tic suppression, and urge intensity ratings remained relatively stable within sessions. The authors concluded that there was no indication of within-session habituation. Finally, Wellen et al. (2023) examined 11 adult TS patients in “free to tic” situations versus those under tic suppression and competing response performance situations [45]. In this study, tic suppression did not result in a reduction in premonitory urges for most participants.

The aim of the present study was to extend the previous findings and examine (1) what the development of premonitory urges entails during ERP sessions of tic suppression, and—as a result—(2) whether or not the hypothesis that within-session habituation to premonitory urges is an underlying working mechanism of successful ERP treatment in TS holds, using a data-driven approach. The most important advantage of a data-driven approach is the opportunity to establish latent trajectories of within-session premonitory urge development that best fit the data.

## 2. Materials and Methods

### 2.1. Participants

The current study took place within the scope of a larger research project, in which treatment effects of one-hour ERP sessions were compared to those from two-hour ERP sessions [37]. The data of patients who had one-hour treatment sessions were used for this project (N = 29), and were collected between May 2008 and May 2009. These 29 patients with TS or chronic tic disorder (CTD) according to the Diagnostic and Statistical Manual of Mental Disorders (4th ed.; DSM-IV (APA, Washington, DC, USA, 1994) were referred by neurologists, psychiatrists and general practitioners to an outpatient clinic specialized in the treatment of tic disorders. Patients had a mean age of 17.97 (SD = 12.15) years, with an age range between 7 and 59 years, including both children and adults. Table 1 presents the demographic and clinical characteristics of the patients. Patients gave written informed consent, and in children below age 18, parents gave written consent as well. The exclusion criteria were as follows: a comorbidity of major depression, a psychotic disorder, and mental retardation. The use of medication for TS and CTD was not an exclusion criterion, as long as type of medication and daily dosages were unchanged throughout the study. Inclusion and exclusion criteria were established by experienced clinicians (the first and third author), using a semi-structured interview, in accordance with DSM-IV criteria [47,48].

Eleven patients (37.9%) used the following medication: dopamine −2 antagonists or modulators (N = 5; olanzapine, risperidone, pipamperone), α- adrenergic drugs (N = 1; clonidine), antidepressants (N = 2; moclobemide, fluoxetine) or a combination of two medications (N = 3; codeine and risperidone, aripiprazole and pipamperone, and pimozide and tetrabenazine). There were no significant differences between patients who used medication and those who did not use medication with respect to patient characteristics at baseline.

### 2.2. Measurements

The Anxiety Disorder Interview Schedule (ADIS) and Mini International Neuropsychiatric Interview (MINI) were used to check for comorbidities [47,48]. Furthermore, the Yale Global Tic Severity Scale (YGTSS, specifically the YGTSS total tic score as used in the study of van de Griendt et al. [37,49]) was used to measure tic severity, and the Premonitory Urge for Tics Scale (PUTS) [9] was used to measure the severity of premonitory urges at baseline. For each patient, the most frequently occurring or interfering tics were selected at baseline, and the intensity of corresponding urges was assessed. Up to 7 different urges were included in this study (range: 2–7; mean number per person: 5.0; SD: 1.0). During ten 60 min sessions, therapists asked the patients to rate the intensity of their urges on a five-point Subjective Units of Distress Scale (SUDS) with scores of 0 (absent), 1 (mild), 2 (moderate), 3 (severe) and 4 (very severe). Urges were asked about before starting each ERP session and at 5, 10, 15, 30, 45 and 60 min. During the first quarter, the urges were asked about more frequently to be able to detect a possible initial rise in urges. A total urge intensity score (SUD-score) was obtained (range = 0–28 per timepoint). The main outcome measure was the 0–4 urge severity rating for each individual tic per timepoint.

### 2.3. Treatment

Treatment consisted of 12 weekly, face-to-face 60 min sessions, including two training sessions of response prevention only, in which patients learned to suppress tics for increasing periods of time. The results of the two training sessions are not included in this study, since premonitory urge characteristics were not assessed. In the following 10 ERP sessions, patients were asked to suppress their tics for as long as possible up to one hour, with the additional instruction that they had to focus on the premonitory urges preceding or accompanying tic suppression. Exposure to the premonitory urges was optimized in several ways: for example, patients were asked to concentrate on bodily premonitory urges, and to imagine situations in which they usually have many tics and urges. Also, personalized urge-eliciting parameters were introduced in the session (e.g., exciting games, videotapes of tic programs, etc.). Regardless of the intensity of urges, patients were encouraged to suppress all tics for the duration of the session. Tics that slipped through were counted. Patients were told to practice the suppression of tics at home as well, with the rationale that the more they practiced, the more they would benefit from treatment. Therapists were graduated psychologists in clinical psychology, and all trained on an existing ERP treatment manual [50]. They received regular individual and group supervision by the first and third author, in which videotaped sessions were discussed to enhance treatment integrity.

### 2.4. Statistical Analyses

To describe the study population demographics, standard descriptive statistics were calculated using SPSS (IBM Corp., Armonk, NY, USA, 2013; version 22.0). To test the main hypothesis, that the intensity of premonitory urges would follow an inverted U-shaped curve with a significant reduction in urge intensity within the sessions eventually (within-session habituation), average SUD intensity scores were calculated for each patient at the different time points. All SUD ratings were summed up and divided by the number of ratings at the different points in time during sessions resulting in an average urge intensity score per time point per session. A repeated measures ANOVA (RMA) was performed on the average SUD intensity scores to test if there was a significant decrease in average SUD-scores within sessions. After investigating if the overall effect was significant, a trend analysis (‘Test of Within-Subjects Contrasts’) was performed to test whether or not changes in SUD scores could be explained by either a linear or a quadratic relationship in the data, followed by a quadratic regression analysis to confirm whether or not within-patient SUD-scores during the treatment sessions followed a quadratic habituation trend.

Next, to enhance comparability between patients who reported different numbers of urges associated with their most frequent or interfering tics, confirmative factor analyses (CFA) were executed on the raw SUD intensity data in Mplus (version 6.12, Muthen and Muthen, Los Angeles, CA, USA, copyright 1998–2011). This was performed because SPSS excludes cases with missing values in at least one of the specified variables, excluding every patient who does not report 7 urges. Using CFA, factor scores of the average SUD-scores of patients across sessions were computed, resulting in more reliable scores without missing data. Finally, as a main outcome measure, latent growth modeling (LGM) was executed on these factor scores in Mplus, examining quadratic, linear and piecewise regression models. The quadratic and linear regression models were based on earlier research [38,46], and the piecewise model was based on an expected increase in the first fifteen minutes (in line with hypothesized initial urge activation based on patient reports that urges intensify with tic suppression). Model fits for each model were computed using the following fit indices: the Akaike information criterion and Bayesian information criterion (AIC/BIC; the lower the value, the better the fit), comparative fit index and Tucker–Lewis index (CFI/TLI, where a score above 0.900 (preferably 0.98) is closest to the model fit), the root mean square error of approximation (RMSEA, the lower the value, the better fit, with a score of <0.08 (preferably even < 0.05) is ideal) and the chi square test (where the model fits best when no significant differences are found).

## 3. Results

**Figure 1:**
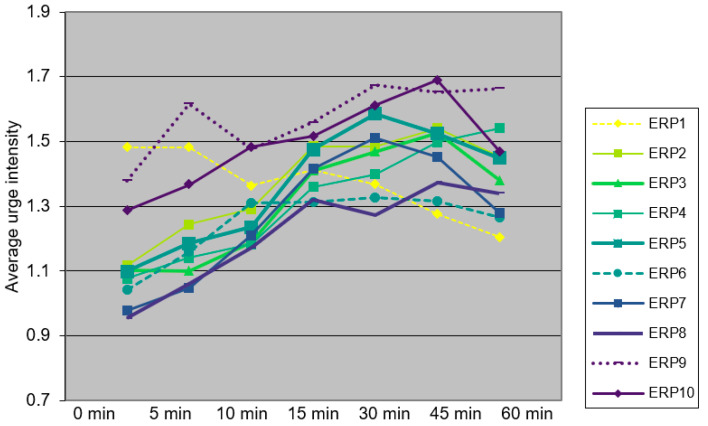
Average urge intensity scores within sessions, averaged across patients (N = 29).

Figure 1 shows the average urge intensities for each ERP session. An inspection of this figure shows that, with the exception of session 1, urge intensity increased during most sessions. An average of 5.7 tics per session slipped through (ranging from an average of 9.6 tics (SD 12.5) in ERP session 1 to an average of 4.7 tics in ERP session 10 (SD 11.3).

**Figure 2:**
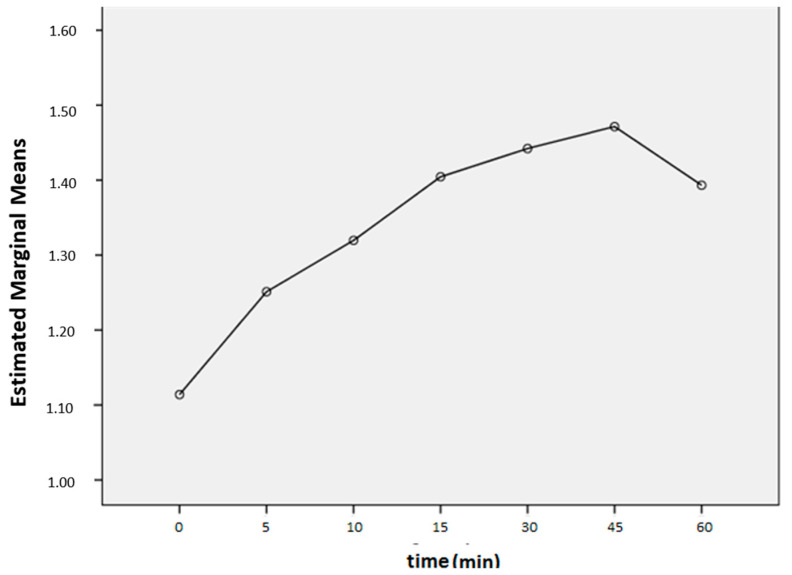
Mean urge intensity scores within sessions, averaged across patients (N = 29) and sessions (N = 10).

To obtain a visualization of the overall course of the premonitory urge intensity of all sessions in all patients (Figure 2), within-session repeated measures analyses (RMA) were performed, in which urges were averaged across patients and sessions. Repeated measures analyses revealed an overall increase in urge intensity during each of the sessions (*F* (1.49, 41.63) = 5.81, *p* < 0.05).

According to the test of within-subjects contrasts, there was a significant linear effect, *F* (1, 28) = 5.50, *p* < 0.05, as well as a significant quadratic effect, *F* (1, 28) = 14.71, *p* < 0.01.

As a next step, confirmative factor analyses scores in the average urge scores per moment in time were used in a latent growth model, testing a linear, quadratic and a piecewise model. In Table 2, the results of this latent growth modeling can be found.

**Figure 3:**
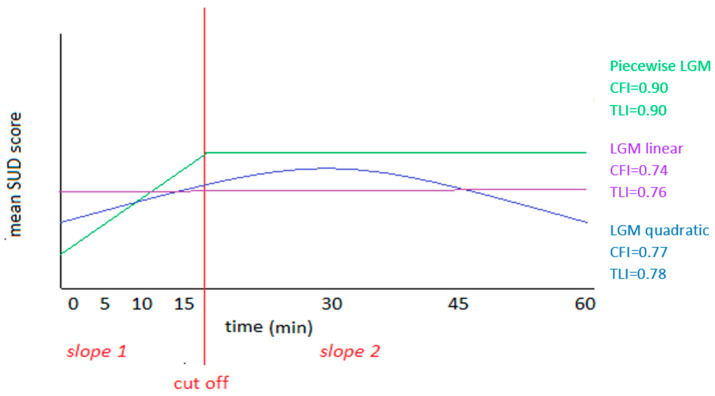
Graphic representation of LGM linear, quadratic and piecewise model.

The piecewise model showed the lowest AIC/BIC values, and the highest CFI/TLI scores, indicating the best model fit of the three tested models. Figure 3 presents a summarizing graph of the slopes of each tested model, showing that in the piecewise model a significant increase in premonitory urge intensity occurred during the first 15 min of each session, remaining stable at this increased level for the remaining 45 min.

## 4. Discussion

This relatively large-scale study tested the course of the intensity of premonitory urges during ERP sessions aimed at suppressing tics while exposing participants to premonitory urges, using a data-driven approach. We found that tic suppression during sessions induced an initial increase in premonitory urge intensity, that was maintained throughout the session. From earlier research, we know that tic severity diminished in this group in the course of sessions, with mean scores of 18.8 (SD 7.0) at baseline, to 12.2 (SD 6.8) after treatment [37]. On average, the initial rise in the intensity of urges during the first 15 min of sessions stabilized for the remaining 45 min at this increased intensity level. The core assumption has been that, by exposing patients for a prolonged period of time to premonitory urges while suppressing tics, these urges will decrease in intensity (a process of habituation) and—as a consequence—tic frequency and severity will decrease. Although we were not able to directly relate our premonitory urge findings with the change in tic severity within sessions because the YGTSS was not measured every session, the results of this study did not support the assumption of a direct relation between exposure to premonitory urges and within-session habituation to premonitory urges for tics.

The course of premonitory urge intensity in this study differs from the linear within-session reduction found in the study of Verdellen et al. [38] and is only partly in line with the results found in the study of Specht et al. [46], who found high and stable urge intensity ratings without an initial increase. A recent study by Wellen et al. [45] also showed no evidence of within-session habituation; tic suppression did not result in a reduction in premonitory urges. In our study, a small, non-significant decline in urges could be seen in the RMA analyses after 45 min, but this effect disappeared in the LGM analysis. One could ask oneself whether or not this decrease would have continued with a longer session duration. On the other hand, the fact that a patient knows that the session is almost ending (and tics can be released again) could have influenced the urge as well. In that case, a longer session duration would only postpone this slight decrease.

Since the habituation hypothesis could not be supported, other underlying working mechanisms should be considered to have played a role in the perceived tic reductions. The presumed linear positive relationship between tics and premonitory urges seems debatable. Our findings are in line with recent doubts about the strong coupling between premonitory urges and subsequent tic execution. In children, the onset of tics mostly precedes the development of premonitory urges [5], going against the idea of a direct stimulus–response relationship between the two. Furthermore, a neuro-imaging study by Ganos and colleagues (2012) indicated no direct relation between tic inhibition and premonitory urge intensity [51]. They stated that changes in those inhibitory neural pathways that control tics are not necessarily accompanied by changes in the (independent) neural pathways that control the premonitory urges. Similarly, Brandt et al. (2016) found support for a degree of independence between urge intensity and tic frequency during voluntary tic suppression. Changes in urge intensity seem to be partly independent of the execution of tics, while executing a tic was still associated with a relief in urge intensity [12]. In a recent study by Wellen et al. (2023), about half of the patients showed relief following tics and not all tics led to immediate relief. Sometimes, bouts of tics were needed to decrease the urge [45].

Tic treatment is originally based on the hypothesis of interrupting a negative reinforcement cycle (urge → tic → relief), for which some experimental support has been found [11,45]. However, as described in the studies above, there is considerable heterogeneity in the urge–tic relationship and there are indications that the urge is -partly- independent of the tic itself, challenging the existence of a negative reinforcement cycle, at least in some individuals. Possibly, the relationship between tics and urges is based on instrumental learning (tic → relief), where the tic is relieving in itself and thus leads to a repetition of the tic, while the urge is initially not part of the instrumental learning cycle. By suppressing the tic during ERP (or, likewise, in HRT, by preventing the tic by executing a competing response), relief does not take place, and the instrumental learning cycle is interrupted.

Alternative underlying working mechanisms can be formulated as well. For example, inhibitory learning could play a role; besides the original pathway (urge- tic), a new pathway develops (urge-no tic), which will strengthen over time with practicing ERP or HRT. Tic inhibition in the presence of high levels of urges, as shown in our study, might even (consciously or unconsciously) strengthen this new pathway, leading to urge tolerance. However, this model also puts a lot of emphasis on the urge as an essential factor. Some aspects of the inhibitory learning model fit cognitive models that use behavioral experiments to disconfirm beliefs and assumptions. In the treatment of tic disorders, patients learn that they are able and competent to control their tics (even when urges are high). Steinberg and colleagues found strong positive correlations between tic-related cognitions and premonitory urges [52]. Likewise, Nissen et al. (2019) found significant reductions in the Beliefs About Tics Scale (BATS, [52]) after a combined ERP/HRT treatment, suggesting that tic treatment had a significant positive impact on dysfunctional cognitions and interpretations related to the necessity to perform a tic. O’Connor (2002) has described a cognitive psychophysiological model in which negative appraisals of tics can locally reinforce tic onset, also suggesting a direct relationship between cognitions and tic execution, independent from premonitory urges [53]. According to Essoe and colleagues (2021), most evidence can be found for an interaction between associative learning and cognitive control, with differences between youth and adults [54]. Where tic-specific inhibition/suppression seems to play a role in adults, more cognitive control processes seem to play a role in youth. Finally, Gagné (2019) has introduced a comprehensive integrated model, including various biological, behavioral, emotional and cognitive factors, with both positive and negative beliefs influencing urges and tics, as well as positive and negative reinforcers that potentially maintain this process [55]. For future treatment, more emphasis on dysfunctional cognitions by examining and actively challenging “harm expectancies” before and after treatment sessions may increase treatment effects.

To conclude, there seems to be no “one-size-fits-all” explanation for the underlying mechanisms of behavioral treatment in tic disorders. Overall, diminishing the urge to tic does not seem to play a crucial role in decreasing tic frequency and intensity during treatment. With this information, we can fine-tune the rationale we give to patients in clinical practice. For example, in ERP, patients can discover that they can control their tics (even with very high urges), and in HRT, patients can learn to conduct a competing response as long as they need it (instead of conducting a competing response for a minute or until the urge goes away).

There are some limitations to this study that need mentioning. We only included a subjective measurement of urge intensity. The addition of an objective measurement of physical arousal would add information to the subjectively reported SUD scores. However, at present, there is no objective instrument available for measuring premonitory urges. Secondly, the study population consisted of 29 participants, which is a relatively small sample size, with relatively mild symptoms based on the YGTSS and comorbidity at start of treatment, giving some room to outliers with divergent response tendencies to influence the current results. However, from each participant there is information on 10 sessions and seven measurement moments per session of a maximum of seven urges, which significantly enhances the power to detect meaningful profiles. Moreover, the use of CFA and LGM in Mplus, besides SPSS, takes into account the sample size and gives more robustness to the findings. Additionally, although the sample had relatively mild symptoms according to the YGTSS, the premonitory urges according to the PUTS at baseline were moderate (19.9 on a scale from 9 to 36) and comparable to those in other studies [21,56,57]. This leaves enough room for change in urge intensity over time. The SUD scores at baseline are also comparable to those of another treatment study [38]. A third limitation is that we did not assess tic severity before and directly after each treatment session, which has limited directly testing the associations between premonitory urge intensity and tic severity. From a research point of view, tic severity and urge intensity seem to be correlated [9], raising the question of whether or not the results are generalizable to a population with more severe tics or stronger urges. Fourth, the continuation of paying attention to the urges, as is one of the key elements of ERP, may have artificially increased the perceived intensity of the urge. The rise in urges was found at 15 min where an urge was rated every 5 min, while stabilization was found when the urge was rated every 15 min. Possibly, asking for urges may trigger the urge itself. Finally, we did not investigate the within-session severity of premonitory urges in the course of the 10 ERP sessions, but lumped all sessions together to study within-session urge intensity patterns. As a consequence, we were unable to investigate whether or not urge intensity diminishes between sessions.

## 5. Conclusions

Within-session premonitory urge intensity follows a piecewise model in course, suggesting that a within-session decrease in urge intensity is not a prerequisite for treatment success. The current study has provided us new information on the link between the within-session intensity of premonitory urges and ERP for tics, which can be directly used to fine-tune the rationale we give to patients in clinical practice. Within-session habituation to premonitory urges was not supported as an underlying working mechanism of the effect of ERP on tic reduction in TS. Rather, other mechanisms including instrumental learning, inhibitory learning and cognitive reappraisal may be at play and deserve further study. Further experimental research into the neurobiological association between premonitory urges and both the inhibition and execution of tics can give new information to further optimize ERP and behavioral therapy for tics.

## Figures and Tables

**Table 1:** Characteristics of the patient sample (N = 29).

		N (%)	Mean (SD)
Sex	Male	20 (69%)	
	Female	9 (31%)	
Age			17.97 (12.15)
Diagnosis	TS	26 (89.6%)	
	CTD	3 (10.4%)	
Age of onset			6.59 (2.48)
Illness duration (yrs)			9.07 (8.13)
YGTSS Total Tic Score at baseline			18.82 (7.01)
PUTS at baseline			19.9 (5.6)
Comorbidity	None	25 (86.2%)	
	ADHD	1 (3.4%)	
	Intermittent Explosive Disorder	1 (3.4%)	
	GAD	2 (6.9%)	
Medication	No	18 (62.1%)	
	Yes	11 (37.9%)	

Notes: TS: Tourette syndrome; CTD: chronic tic disorder; YGTSS: Yale Global Tic Severity Scale [49]; PUTS: Premonitory Urge for Tics Scale [9]; ADHD: attention deficit hyperactivity disorder; GAD: generalized anxiety disorder; SD: standard deviation.

**Table 2:** Results of latent growth modeling.

	AIC/BIC	CFI	TLI	RMSEA (Probability ≤ 0.05)	Chi Square (Degrees of Freedom)	Significance Chi Square
Linear model	37.72/54.13	0.74	0.76	0.40	128.59 (23)	0.00
Quadratic model	28.27/46.05	0.77	0.78	0.39	117.14 (22)	0.00
Piece wise model	−25.31/−6.17	0.90	0.900	0.26 (0.00)	61.56 (21)	0.00

Notes. N = 29; AIC = Akaike information criterion; BIC = Bayesian information criterion; CFI = comparative fit index; TLI = Tucker–Lewis index; RMSEA = root mean square error of approximation.

## Data Availability

Data are contained within the article.
